# Psychological and psychosocial determinants of COVID Health Related Behaviours (COHeRe): An evidence and gap map

**DOI:** 10.1002/cl2.1336

**Published:** 2023-06-22

**Authors:** Jennifer Hanratty, Ciara Keenan, Sean R. O'Connor, Rachel Leonard, Yuan Chi, Janet Ferguson, Ariana Axiaq, Sarah Miller, Declan Bradley, Martin Dempster

**Affiliations:** ^1^ School of Psychology Queen's University Belfast Belfast UK; ^2^ Centre for Effective Services Belfast UK; ^3^ Campbell UK & Ireland Belfast UK; ^4^ Cochrane Global Ageing Shanghai China; ^5^ Applied Behaviour Research Clinic University of Galway Galway Ireland; ^6^ School of Education, Social Sciences and Social Work Queen's University Belfast Belfast UK; ^7^ Centre for Public Health Queens University Belfast UK

## Abstract

**Background:**

The COVID‐19 pandemic, caused by the SARS‐CoV‐2 virus, has resulted in illness, deaths and societal disruption on a global scale. Societies have implemented various control measures to reduce transmission of the virus and mitigate its impact. Individual behavioural changes are crucial to the successful implementation of these measures. Common recommended measures to limit risk of infection include frequent handwashing, reducing the frequency of social interactions and the use of face coverings. It is important to identify those factors that can predict the uptake and maintenance of these protective behaviours.

**Objectives:**

We aimed to identify and map the existing evidence (published and unpublished) on psychological and psychosocial factors that determine uptake and adherence to behaviours aimed at reducing the risk of infection or transmission of COVID‐19.

**Search Methods:**

Our extensive search included electronic databases (*n* = 12), web searches, conference proceedings, government reports, other repositories including both published peer reviewed, pre‐prints and grey literature. The search strategy was built around three concepts of interest including (1) context (terms relating to COVID‐19), (2) behaviours of interest and (3) terms related to psychological and psychosocial determinants of COVID Health‐Related Behaviours and adherence or compliance with recommended behaviours, to capture both malleable and non‐malleable determinants (i.e. determinants that could be changed and those that could not).

**Selection Criteria:**

This Evidence and Gap Map (EGM) includes all types of studies examining determinants of common recommended behaviours aimed at mitigating human‐to‐human spread of COVID‐19. All potential malleable and non‐malleable determinants of one or more behaviours are included in the map. As part of the mapping process, categories are used to group determinants. The mapping categories were based on a previous rapid review by Hanratty 2021. These include: ‘behaviour’, ‘cognition’, ‘demographics’, ‘disease’, ‘emotions’, ‘health status’, ‘information’, ‘intervention’, and ‘knowledge’. Those not suitable for categorisation in any of these groups are included in the map as ‘other’ determinants.

**Data Collection and Analysis:**

Results were imported to a bibliographic reference manager where duplications of identical studies gathered from multiple sources were removed. Data extraction procedures were managed in EPPI‐Reviewer software. Information on study type, population, behaviours measured and determinants measured were extracted. We appraised the methodological quality of systematic reviews with AMSTAR‐2. We did not appraise the quality of primary studies in this map.

**Main Results:**

As of 1 June 2022 the EGM includes 1034 records reporting on 860 cross‐sectional, 68 longitudinal, 78 qualitative, 25 reviews, 62 interventional, and 39 other studies (e.g., mixed‐methods approaches). The map includes studies that measured social distancing (*n* = 487), masks and face coverings (*n* = 382), handwashing (*n* = 308), physical distancing (*n* = 177), isolation/quarantine (*n* = 157), respiratory hygiene/etiquette (*n* = 75), cleaning surfaces (*n* = 59), and avoiding touching the T‐zone (*n* = 48). There were 333 studies that assessed composite measures of two or more behaviours. The largest cluster of determinants was ‘demographics’ (*n* = 730 studies), followed by ‘cognition’ (*n* = 496 studies) and determinants categorised as ‘other’ (*n* = 447). These included factors such as ‘beliefs’, ‘culture’ and ‘access to resources’. Less evidence is available for some determinants such as ‘interventions’ (*n* = 99 studies), ‘information’ (*n* = 101 studies), and ‘behaviour’ (149 studies).

**Authors' Conclusions:**

This EGM provides a valuable resource for researchers, policy‐makers and the public to access the available evidence on the determinants of various COVID‐19 health‐related behaviours. The map can also be used to help guide research commissioning, by evidence synthesis teams and evidence intermediaries to inform policy during the ongoing pandemic and potential future outbreaks of COVID‐19 or other respiratory infections. Evidence included in the map will be explored further through a series of systematic reviews examining the strength of the associations between malleable determinants and the uptake and maintenance of individual protective behaviours.

## 1 PLAIN LANGUAGE SUMMARY

### 1.1 Most COVID‐19 studies looked at the influence of sex, age and our perception of COVID‐19 on social distancing, handwashing and face covering

Over the course of the COVID‐19 pandemic there have been huge volumes of research looking at how different factors influence COVID‐19 health protective behaviours: social distancing, face covering and handwashing. The majority of this research is focused on factors that cannot be altered through intervention, such as demographics (age and sex).

### 1.2 What is this evidence and gap map (EGM) about?

In the early stages of the COVID‐19 pandemic, health protective behaviours such as distancing and handwashing were the first line of defence to help limit the spread of COVID‐19. Understanding why people do or do not engage in recommended behaviours is important to developing successful public health messaging, and to increase the number of people engaging in these behaviours.

This EGM summarises studies that measured one or more factors that might influence health protective behaviours, including handwashing, use of face coverings, social or physical distancing, and isolation or quarantine.

**What is the aim of this EGM?**

The aim of this EGM is to map the current research on different factors and health protective behaviours, such as social distancing, handwashing and face covering.

### 1.3 What studies are included?

The current map includes 1,034 pieces of evidence. This consists of 25 reviews (including 17 systematic reviews) and 1,009 primary studies. Of these, 860 were cross‐sectional studies, 68 were longitudinal, and 78 were qualitative studies.

### 1.4 What are the main findings of this EGM?

There was lots of evidence on some behaviours (social distancing, handwashing, face covering) and very little on others (not touching your face, cleaning surfaces).

Social distancing had the most evidence (487 studies), followed by use of face coverings (382 studies) and handwashing (308 studies). Fewer studies looked at behaviours such as avoiding the T‐zone (not touching your face), cleaning surfaces and respiratory hygiene practices (coughing into your elbow or tissue).

A large number of studies (333 studies) combined two or more protective behaviours within the one study. This makes it difficult to use these studies to look at how different factors may influence individual behaviours.

Across the studies there are significant differences in how different behaviours are defined. Some studies described social distancing as minimising contacts outside of the home, whereas others used the term to mean physical distancing from others (for example, keeping at least two metres apart).

In relation to the different factors influencing these behaviours, demographics was the most reported (730 studies), followed by cognitive factors (how people might perceive or think about COVID‐19; 496 studies).

Fewer studies looked at the influence of factors such as interventions (for example, providing access to hand sanitiser), information on COVID‐19 (for example, the different sources of information people received) and the impact of engaging in other protective behaviours (for example, if someone covered their face, would they be more or less likely to also social distance).

### 1.5 What do the findings of the map mean?

This EGM shows the available evidence on the different factors that may influence health protective behaviours such as distancing, face coverings and handwashing.

This EGM can be used to help guide future areas of research and public health policy during the current COVID‐19 pandemic and any future outbreaks of respiratory viruses with pandemic potential.

Current evidence is largely focused on social distancing and the use of face coverings; and primarily on the influence of factors such as age or sex (demographics) on these behaviours. There are major gaps in the evidence base on other important health protective behaviours and the influence of factors other than age or sex.

### 1.6 How up‐to‐date is this EGM?

The authors searched for studies up to October 2021.

## 2 BACKGROUND

### 2.1 Introduction

#### 2.1.1 The problem, condition or issue

Severe acute respiratory coronavirus 2 (SARS‐CoV‐2) emerged in late 2019 and spread rapidly around the globe (Cucinotta et al., 2020; Wu et al., 2020). The pandemic of COVID‐19 disease, caused by SARS‐CoV‐2, has resulted in short and long‐term illness, deaths and societal disruption. Societies implemented control measures to reduce the transmission of the virus. Various recommended behaviours such as, handwashing or use of hand sanitiser, wearing masks or face coverings, physical distancing, social distancing, isolation or quarantine, respiratory hygiene, cleaning surfaces, avoiding touching the ‘T‐zone’ (mouth, nose and eyes) (Elder et al., 2014), were introduced globally to reduce the risk of catching or spreading SARS‐CoV‐2. The evidence for the effectiveness of these measures has been established during previous pandemics of similar serious viral respiratory infections such as pandemic Influenza A (H1N1), SARS and MERS (Flumignan et al., 2020; Jefferson et al., 2020; Seto et al., 2003; Warren‐Gash 2013; Webster et al., 2020; West et al., 2020). While all of these behaviours were recommended at the outset of COVID‐19, these have changed over the course of the pandemic.

Individual behaviour change is crucial to the success of these measures through reducing the frequency of social contacts, mitigating the risk of those social contacts and reducing the amount of time that infectious people are in contact with others whom they may infect. Vaccine programmes were introduced in December 2020 but even in this context, with waning immunity and the evolution of new variants, behavioural measures to reduce the spread remain vital (Girum et al., 2021; Michie et al., 2020). It is important to synthesise evidence from the COVID‐19 pandemic that may be applied to future pandemics of influenza and other serious respiratory infectious diseases.

#### 2.1.2 Why it is important to develop the Evidence and Gap Map (EGM)

Health protective behaviours cannot be effective on a societal level if they are not adopted widely and consistently. Variables such as individual health beliefs, social support, culture and social norms can all influence the likelihood of someone undertaking and maintaining particular health behaviours. To develop appropriate public health interventions to improve uptake and adherence to these behaviours, including effective messaging, it is important to understand the malleable factors that influence these behaviours. We identified and mapped all existing research evidence that described a relationship between any factor or determinant and health‐protective behaviours in the context of SARS‐CoV‐2.

In this EGM we are interested in the evidence on malleable and non‐malleable psychological and psychosocial factors associated with uptake and adherence to health protective behaviours. Malleable determinants in this EGM refer to psychological and psychosocial factors that can be developed, shaped or altered. Factors such knowledge, access to information, emotions, and perceptions. Non‐malleable determinants in this EGM refer to factors or attributes that are fixed or unchangeable through public health intervention. Factors such as age, sex, income, past behaviour and health status.

In any future severe viral outbreaks, health‐protective behaviours will be vital to reducing risk of infection and transmission. Non‐pharmaceutical interventions that are designed to improve the uptake and adherence to protective behaviours are essential in an outbreak, and in particular when vaccines and treatments are not yet established. The effectiveness of these behaviour change interventions will be determined, to some extent, by how they address the psychological and psychosocial variables that influence behaviour. To optimise public health intervention, we need to know which specific variables are most likely to influence the target behaviours in this context. Evidence gathered in the context of COVID‐19 can inform who, when and under what circumstances people do or do not adopt recommended preventive behaviours.

There has been an exponential increase in the volume of studies conducted in the context of COVID‐19 when compared to previous similar outbreaks. Our recent rapid review of studies conducted in the context of other similar outbreaks (Pandemic influenza, SARS, MERS and others; Hanratty et al., 2021) identified 58 studies that met the same inclusion criteria as the current EGM. That's 58 studies published over decades compared to 383 studies produced in 2020 and 624 in 2021).

#### 2.1.3 Existing EGMs and/or relevant systematic reviews

We searched COVID‐END inventory of evidence synthesis and identified a number of EGMs related to COVID‐19 treatments, the effectiveness of control measures and the impact of COVID, however, we have not been able to identify any other EGM that maps evidence on determinants of uptake and adherence to protective behaviours.

There are a number of related published and ongoing reviews on individual determinants of COVID‐19 health‐related behaviours but none with the broad scope of this EGM. Using robust search, retrieval, and methodological approaches to minimise potential sources of bias, this EGM summarises the existing and emerging evidence on determinants of health‐protective behaviours in the context of the COVID‐19 pandemic.

## 3 OBJECTIVES

We aimed to identify and map the existing evidence (both published and unpublished) on malleable (those that can be most effectively targeted as part of public health interventions) and non‐malleable psychological and psychosocial factors associated with uptake and adherence to behaviours that can reduce the risk of infection or transmission of COVID‐19 (West et al., 2020). The specific behaviours of interest were as follows:

- Handwashing
- Wearing masks/face coverings
- Physical Distancing
- Social Distancing
- Isolation/quarantine
- Respiratory hygiene
- Cleaning surfaces
- Avoiding t‐zone
- Other composite measures that include the above.

The EGM provides an initial step of a wider project (https://www.qub.ac.uk/schools/psy/Research/OurResearchThemes/HealthWelfareClinicalPsychology/COHeRe/) which will also examine evidence included in the map further through a series of systematic reviews examining which malleable determinants (or those that can be most effectively targeted as part of public health interventions) are more closely associated with uptake and maintenance of individual protective behaviours.

## 4 METHODS

### 4.1 EGM: Definition and purpose

EGMs are a tool to support researchers, funders and evidence intermediaries to identify what research exists and where there are gaps in a particular research area. EGMs are a powerful tool to support evidence‐informed practice and policy decisions Miake‐Lye (2016); White et al. (2020).

In producing this EGM we followed five steps:

(1) Scoping and development of the EGM framework
(2) systematic and comprehensive searches
(3) screening for eligibility (i.e., title & abstract, then full text)
(4) data extraction
(5) high‐level snythesis of included evidence reviews (according to the predefined inclusion/exclusion criteria)

### 4.2 Conceptual framework

We deliberately did not approach this EGM with a particular framework or theory of health behaviour driving the selection of potential determinants or synthesis decisions. Instead, we sought to identify separate groups of non‐malleable and potentially malleable determinants that could be influenced by public health interventions. To develop an EGM framework that best represented research on determinants of COVID‐19‐related behaviours, the specifics of the framework were developed in consultation with key stakeholders involved in the advisory groups. This framework formed the basis of the data extraction for visual presentation of the included evidence, with rows representing the behaviours of interest measured and columns representing the determinants of behaviour measured. The map also included information filters such as the study design, sector and age of participants.

#### 4.2.1 Types of studies

The EGM had broad eligibility criteria and included all study designs that presented data on a relationship between any potential determinant and one or more of the behaviours of interest. We included observational studies (both retrospective and prospective), experimental studies, systematic reviews and qualitative studies. We included studies that measured non‐malleable determinants, such as demographics and malleable determinants such as knowledge of the disease, and perceived risk to health.

#### 4.2.2 Population

The population of interest is members of the public, of any age. We included studies on the general public as well as specific groups that may be at increased risk of exposure for example, people who work in essential retail services. Similarly, we included studies of specific patient groups at increased risk of becoming seriously ill if infected, for example, those with chronic respiratory disorders.

We did not include studies on health care workers (HCWs), defined as someone who works in a hospital or health care setting or who delivers health care in the community. This population typically have additional knowledge, training and resources to support the adoption of behaviours to mitigate against the increased risk of exposure to infectious diseases. A rapid review on barriers and facilitators to HCWs adherence to infection prevention and control guidelines has been published (Houghton et al., 2020). For studies that include both HCWs and the public, these are only included in the map if data is presented separately for the general public.

#### 4.2.3 Context

We included studies which were conducted during the ongoing COVID‐19 pandemic and therefore only included studies between Jan 2020 to the date of the final search.

#### 4.2.4 Exposure/Determinants

The exposure in this EGM refers to any potential determinant of one or more of the behaviours of interest described above. Within the EGM this included both malleable and non‐malleable determinants.

We developed 10 categories of determinants for the mapping process. These included, behaviour, cognition, demographics, disease, emotions, health status, information, intervention, knowledge and other. Each category was divided into subcategories of various determinants (Table 1). A description of each individual category and subcategory is available in Supporting Information: Appendix 1.

**Table 1:** Determinant by category and subcategory.

Behaviour	Cognition	Demographics	Disease	Emotions	Health Status	Information	Intervention	Knowledge	Other
Past behaviour	Behaviour	Age	Status	Disease	General	Info seeking/consuming	Education	Behaviour	Beliefs
Intention	Disease	Education	Proximity	Other	At risk group	Quality/source	Info campaign	Disease	Social
Other protective behaviour	Motivation	Employment	Other		Vaccine status	Messaging	Other	Other	Resources
	Other	Ethnicity			Other				Other
	Social	Geographic location			Disability				Time
	Cognitive capacity	Parental status							Cultural
		Relationship status							
		Religion							
		SES							
		Sex							
		Other							
		Multi‐generational households							
		Caring responsibilities/carers							

**Behaviour** captured determinants that related a persons health‐protective behaviour to past behaviour, behavioural intentions or other protective behaviours.

**Cognition** was broken down into six subcategories: thoughts or perceptions about the protective behaviours; about COVID‐19; motivations; social cognition (e.g. perceived social norms); cognitive capacity indicating a person's ability to understand or retain information; ‘other’ to capture any other cognitive determinant that did not fit into the previous five subcategories.

**Demographic** determinants included; age, education, employment, ethnicity, geographic location, parental status, relationship status, religion, socioeconomic status, sex, multi‐generational household, caring responsibilities and other, which was used to capture other determinants that did not fit into the previous subcategories (e.g., political affiliation).

**Disease** captured determinants relating to COVID‐19 including infection status such as positive test or having symptoms; proximity to the disease for example local case rates; and ‘other’.

**Emotions** captured determinants related to feelings about the disease and ‘other’ emotion‐related determinants for example general emotional state or mood.

**Health status** included the subcategories of general health, at‐risk groups, which included those with mental health problems, vaccine status, and those with disabilities.

**Information** included seeking and consuming information, the quality or source of information, and determinants related to public health messaging for example message content or framing.

**Interventions** were categorised and divided into educational interventions, information campaigns and ‘other’ types of relevant interventions.

**Knowledge** included determinants relating to knowledge about protective behaviours, knowledge about the disease and any other types of assessed knowledge, such as knowledge of regulations or knowledge of vaccines.

**Other** was the final category of determinants and includes any determinants that did not fit within the previous broad categories. This was divided into subcategories of beliefs for example political beliefs, social (e.g. social capital, social networks), practical resources such as access to masks, paid sick leave, time included time since the outbreak began, cultural determinants such as collectivist vs individualist cultures, and a final ‘other’ subcategory for any remaining determinant that did not fit into the previous subcategories.

#### 4.2.5 Measurement of determinants

We included studies that measured determinants at an individual level and group level, for example, country‐level data on the number of cases.

We included studies on self‐reported or observed determinants. Self‐reports included actual or perceived determinants, for example ‘risk of contracting the virus” could be measured by quantifying actual risk based on individual circumstances and behaviour or through self‐reported perceived risk.

Studies that did not present data on the relationship between a potential determinant and one or more of the behaviours of interest were excluded from the map.

#### 4.2.6 Outcomes: Behaviours of interest

This EGM sought to map evidence on determinants of the commonly recommended behaviours to mitigate human‐to‐human spread of COVID‐19 as described by  West et al. (2020). Table 2 describes in detail the behaviours of interest in this EGM.

**Table 2:** Behaviours to mitigate the spread of COVID‐19.

Behaviours	Description of behaviours
**Handwashing**	Washing hands more frequently with soap and water or the use of hand sanitizer if handwashing facilities are not available
**Masks/face covering**	Wearing any type of mask or face covering. This can include medical grade masks, face shields, homemade masks or covering face with a scarf.
**Physical Distancing**	Maintaining the recommended distance from others when physically present. The recommended distance varies by setting but is typically in the region of 1 to 3 m.
**Social Distancing**	Minimising social contact with those outside of your own household. This is a very broad category and includes working from home, avoiding crowded places, only leaving home when necessary (e.g. to purchase food or medicines) and not socialising with others in your own home or garden.
**Isolation/quarantine**	Self‐isolation and/or quarantine refers to keeping separate from all other people either because you have or are suspected to have the virus. Self‐isolation is typically voluntary but often recommended by the government/health authorities. Quarantine is typically enforced in either a mandated setting, one's own home, or temporary accommodation for those in travelling away from home.
**Respiratory hygiene**	Includes tissue hygiene, which means using a tissue to cover nose and mouth when coughing, sneezing or blowing your nose and immediately disposing of the tissue. When tissues are not available coughing/sneezing into your elbow and not your hands.
**Cleaning surfaces**	Disinfecting high touch surfaces in home and office/retail/public spaces or items brought into the home.
**Avoiding T‐zone**	Avoiding touching your face specifically the T‐zone; eyes, nose & mouth
**Other**	Other analogous relevant behaviours or aggregate measures of multiple relevant behaviours

Other behaviours may be recommended in different countries/regions and so the behaviours of interest, listed in Table 2, was not an exhaustive list of behaviours that might mitigate the spread of COVID‐19. They are, however, commonly recommended behaviours globally.

Within the EGM we did not include behaviours such as testing or vaccine uptake for two main reasons. Firstly, evidence on these behaviours in relation to COVID‐19 was not available at the inception of this study as testing was not initially widely available and vaccines were yet to be developed. Secondly, vaccines and testing are unlikely to be available in the initial days, weeks and months of any future pandemic. Therefore, we opted to focus on behaviours that could be adopted without delay in the context of COVID‐19 and any future outbreaks of serious respiratory viruses.

#### 4.2.7 Measurement of behaviours

We included studies on actual behaviour, intended behaviour or hypothetical behaviour. We included behaviours measured through self/other report and/or observation of actual behaviour, measured at both the individual and group level.

### 4.3 EGM protocol

The EGM protocol was published in March 2022 (Hanratty et al., 2022).

### 4.4 Framework development and scope

We followed the standard EGM framework as a matrix, with rows containing the behaviour (i.e., Handwashing, wearing masks/face coverings, physical Distancing) and columns containing information regarding the determinants (i.e., Cognitive factors, affective factors). Filters were also added to the EGM. The framework and filters used were first developed using our existing knowledge of the extant evidence gained through our rapid review of the evidence (Hanratty et al., 2021). This first draft of the map framework was constructed using a ‘bottom up’ approach whereby the determinant categories were derived from the determinants measured in studies included in our rapid review (Hanratty et al., 2021), which included studies conducted in the context of COVID‐19 and other previous outbreaks of serious respiratory viral infections (e.g. pandemic influenza, SARS, MERS). The framework was then reviewed by a group of citizen advisors and an expert advisory group (see stakeholder engagement). Both groups provided valuable insights into the structure of the framework, and identified gaps in the framework of determinants that the groups felt were likely to be important but had not been measured in the studies included in the rapid review. For example, cognitive capacity was added after citizen advisors shared their own experiences of caring for family members with limited capacity to understand or retain information. Similarly, multigenerational households did not appear in previous studies as a demographic determinant but citizen advisors agreed that they were likely to modify their behaviour if living with older adults or children attending educational settings. Input from the expert advisory group of policymakers, behaviour change experts and evidence synthesis experts also shaped the map framework. For example, in our rapid review we found few studies that examined particular sectors, such as education, retail settings, or transport, and we intended not to include ‘sector’ as a filter in the EGM, but policymakers made it clear that they wanted that information to be retained, thus allowing us to identify gaps in evidence base relating to sectors.

### 4.5 Search methods and sources

#### 4.5.1 Search methods and sources

Systematic reviews and EGMs are underpinned by a systematic search of the literature, using various literature sources including electronic databases, web searches, conference proceedings, government reports and other repositories of literature. To ensure that the literature contained in the map is relevant and useful to key stakeholders, it was important that the literature retrieval methods followed high‐quality standards and all searches were conducted and reported following Campbell Collaboration guidelines (White et al., 2020).

Information retrieval specialist author (CK) developed and piloted a search strategy with input from clinical and behaviour change expert authors (DB and MD). This strategy was further refined by CK following expert advice from a Campbell information retrieval specialist during the editorial/peer review of the protocol.

The search strategy was built around three concepts of interest:

(1) context (terms relating to COVID‐19). For concept one, we used an innovative and tested COVID‐19 search strategy was developed for use by NICE information specialists and was updated as recently as June 21, 2021 (Levay et al., 2021). An example of the search string was piloted in Medline (Ovid) and is presented in Table 3 below.
(2) behaviours of interest.
(3) terms related to psychological and psychosocial determinants of COVID Health‐Related Behaviours and adherence or compliance with recommended behaviours, to capture both malleable and non‐malleable determinants.

**Table 3:** Medline (Ovid) search strategy.

Appendix 2: Medline (Ovid) search strategy		
Ovid MEDLINE(R) ALL <1946 to 3 September 2021>		
1	SARS‐CoV‐2/or COVID‐19/	103,591
2	(corona* adj1 (virus* or viral*)).ti,ab.	2364
3	(CoV not (Coefficien* or “co‐efficien*“ or covalent* or Covington* or covariant* or covarianc* or “cut‐off value*“ or “cutoff value*“ or “cut‐off volume*“ or “cutoff volume*“ or “combined optimi?ation value*“ or “central vessel trunk*“ or CoVR or CoVS)).ti,ab.	51,911
4	(coronavirus* or 2019nCoV* or 19nCoV* or “2019 novel*“ or Ncov* or “n‐cov” or “SARS‐CoV‐2*“ or “SARSCoV‐2*“ or SARSCoV2* or “SARS‐CoV2*“ or “severe acute respiratory syndrome*“ or COVID*2).ti,ab.	181,470
5	or/1‐4	187,096
6	limit 5 to yr = “2020‐Current”	173,962
7	(6 and english.lg.) not (letter or historical article or comment or editorial or news).pt. not (Animals/not humans/)	134,173
8	(Mask or masks or face?mask* or Face cover*).ti,ab.	42,975
9	(face adj2 (shield or shields)).ti,ab.	414
10	(((Hand or hands) adj2 hygiene) or Handwash* or (Wash* adj2 hand*)).ti,ab.	11,132
11	(hand adj1 clean*).ti,ab.	256
12	(hand adj2 saniti*).ti,ab.	683
13	(hand adj2 disinfect*).ti,ab.	783
14	Respiratory hygiene.ti,ab.	79
15	Respiratory etiquette.ti,ab.	27
16	((cough* or sneeze*) and (sleeve or arm or elbow or tissue or etiquette)).ti,ab.	2752
17	(tissue and (dispose or disposal or bin or hygiene)).ti,ab.	3414
18	universal hygiene.ti,ab.	10
19	Social Isolation/or Patient Isolation/	19,284
20	(self‐isolate or self‐isolation or self‐isolating).ti,ab.	724
21	(mass adj2 (behav* or gather*)).ti,ab.	1690
22	(social distance or social distancing).ti,ab.	6625
23	stay at home.ti,ab.	1465
24	stay home.ti,ab.	314
25	((work* adj2 home) or telecommute or telework* or (remote* adj2 work*)).ti,ab.	5262
26	(Physical adj2 distanc*).ti,ab.	2595
27	(touch* and (mouth or mouths or face or faces or nose or noses or t‐zone)).ti,ab.	1635
28	disinfect*.ti,ab.	31,760
29	lockdown.ti,ab.	8167
30	quarantine.ti,ab.	7821
31	(nonpharmaceutical or non‐pharmaceutical).ti,ab.	1831
32	(school closure or close school* or school closing).ti,ab.	389
33	or/8‐32	140,404
34	limit 33 to yr = “2020‐Current”	34,955
35	(34 and english.lg.) not (letter or historical article or comment or editorial or news).pt. not (Animals/not humans/)	31,455
36	7 and 35	20,298
37	exp Knowledge/	12,323
38	exp Health knowledge, Attitudes, Practice/	119,567
39	(Knowledg* or Personal* or Attitude* or Practice* or Habit* or belie* or Behav* or Need* or prevent* or Compliance or comply* or complied or Perception* or Protect* or Predict* or view* or barrier* or facilitator* or readiness or prepar* or ability* or insight or proficien* or procedur* or adher*).ti,ab.	10,617,318
40	or/37‐39	10,635,825
41	7 and 35 and 40	14,859

For concept 2 and 3 the terms used were based on those used in the rapid review which itself was informed through consultation with the Behaviour Change Group formed in response to COVID‐19 by the Public Health Agency, Northern Ireland. The terms were then piloted and refined in two databases, with unique terms added and redundant or duplicate terms removed (Table 3).

##### Electronic databases

Based on the Queens's University Belfast database subscriptions, we searched the following key information sources to locate relevant primary research:

- Medline ALL (Ovid)
- Child Development & Adolescent Studies (EBSCOhost)
- ERIC (EBSCOhost)
- PsycInfo 1806‐present (OVID)
- CINAHL Plus (EBSCOhost)
- Web of Science Core Collection (the QUB subscription includes SCI‐expanded, SSCI, A&HCI, CPCI‐S, CPCI‐SSH, ESHI)

To locate relevant secondary research for inclusion in the EGM, we searched the following information resources:

- The Social Care Institute for Excellence (SCIE)
- The Cochrane Library
- Epistemonikos Covid‐19 evidence platform
- Norwegian Institute of Public Health living maps
- EPPI—centre
- COVID‐END

##### Other sources

We searched for Grey literature across multiple sources. Grey literature is that which is not published, not peer reviewed, and not easily accessible. Sources of grey literature are varied and include government reports, privately and publicly funded research, conference proceedings, working papers, and posters. Some grey literature sources are captured in the Web of Science search, these include:

- Conference Proceedings Citation Index‐ Science (CPCI‐S)—1990‐present
- Conference Proceedings Citation Index‐ Social Science & Humanities (CPCI‐SSH)—1990‐present

We attempted to locate additional grey literature by searching sources such as the following:

- Google Scholar (We will search https://scholar.google.com/ using an incognito browser and the following strategy: (coronavirus| ‘2019 nCoV’| ‘2019 novel’| ‘2019 nCoV’| ‘2019 nCoV’| CoV |‘COVID 19’ |COVID19| ‘COVID 19’| ncov |‘SARS CoV2’| ‘SARS CoV 2’|‘severe acute respiratory syndrome Coronavirus 2’) (Psychological|Psychosocial)(behavior|behaviour) we will limit returns by ‘Since 2020’ filter and sort remaining records by relevance. We downloaded the first 1000 articles (which is the upper limit set by Google) using Harzing's Publish or Perish software.
- clinicaltrials.gov
- Isrctn Registry (https://www.isrctn.com/)
- WHO International Clinical Trials Registry Platform (ICTRP) (https://www.who.int/clinical-trials-registry-platform/the-ictrp-search-portal)

And by contacting and reviewing the information of the following key organisations in the UK with proven experience on the topics related to this project:

- King's Fund (https://www.kingsfund.org.uk/)
- National Institute for Health Research (https://www.nihr.ac.uk/)
- NHS Evidence (https://www.evidence.nhs.uk/)

We considered searching ProQuest dissertations and theses, however, we assessed that it was unlikely that any relevant doctoral theses would be complete and available in the timeframe of the virus.

We conducted a search of reference lists of previous reviews and eligible articles to identify any additional studies not identified through the electronic search. Finally, when we compiled a list of included studies, we contacted key experts in the field via email (categorised as ‘key’ if they have published five or more included studies) to ask whether they were aware of any unpublished or ongoing research that might not have been easily accessible to the research team.

To locate additional relevant grey literature for inclusion in the EGM, we searched for ongoing or unpublished reviews via:

- PROSPERO,
- Figshare and the
- Open Science Framework (OSF).

Any ongoing reviews were checked again before completion of the project and if still unpublished were excluded from the map.

##### Search limits

Due to the limited language skills of the review team, we only included studies published in English.

We limited our search to exclude opinion pieces, letters, editorials and unpublished reports in databases where these limits are supported (See Table 3: lines 7 and 35). We did not use database limiters for studies on humans only as we found these limiters excluded a substantial number of potentially relevant papers not indexed as ‘human’ studies. Instead, we have opted to use an adaptation of the Cochrane search filter for human studies (lines 7 and 35).

We included only those studies which were conducted during the ongoing COVID‐19 pandemic. We included studies from Jan 2020 until the date of the final search.

### 4.6 Data collection and analysis

#### 4.6.1 Screening and study selection

Once the database searches had been conducted, results were imported to a bibliographic reference manager (EndNote x9) where duplications of identical studies gathered from multiple sources were removed to avoid duplication of effort.

Following this, screening was completed through the Cochrane Crowd (Noel‐Storr, 2021). This platform provides a mechanism for volunteers to undertake different tasks related to the identification of studies as part of health care evidence reviews. The platform has been shown to have sufficient accuracy in terms of study identification (Gartlehner et al., 2020; Noel‐Storr, 2021; Noel‐Storr et al., 2021a; Noel‐Storr et al., 2021b). It also provides a scalable approach, allowing it to support the screening of rapidly emerging evidence. Following successful completion of a brief training module, screeners were asked to screen titles and abstracts against the eligibility criteria and to identify potential studies as ‘possibly relevant’ or ‘not relevant’. Each record was screened by at least three independent screeners. Where there were any conflicts between screener decisions, these were resolved by members of the core research team.

When this initial screening of titles and abstracts was complete, all potentially relevant records were imported into EPPI reviewer. Members of the research team then screened all potentially relevant studies at full‐text level. At least two reviewers screened each full text, working independently. Disagreements were resolved through discussion with the research team. The research team convened weekly online meetings and maintained communication day to day in an online chat, so all team members had access to decisions and clarifications. All screening decisions and coding of the included studies were documented and are publicly available via the project page on Open Science Framework (https://osf.io/hv5s3/).

#### 4.6.2 Data extraction and management

Data extraction procedures were managed in EPPI‐Reviewer software (Thomas, 2010). Once eligible studies were identified from full‐text screening, one author extracted data. Any studies remaining after full‐text screening, but which were subsequently identified as ineligible during data extraction were listed as ‘excluded’ and the reason for exclusion recorded. To maintain quality and consistency in data extraction a second author checked the data extraction on 20% of included papers, with a greater proportion of records checked in the earlier stage of screening. The two people who completed the data extraction for each study in the sample of 20% discussed any discrepancies until they reached a consensus or, if necessary, referred to a third author to make a final decision. As with full‐text screening, we convened weekly meetings to discuss data extraction and maintained day‐to‐day contact via an online chat to quickly address any questions and share decisions and clarifications.

Data extraction for the EGM was done by coding each study according to each of the categories outlined below. Individual studies could be assigned more than one code in each category, for example a mixed methods study could be coded under study design as ‘qualitative’ and ‘Observational—cross‐sectional’. Similarly, studies that included a wide range of ages were coded according to all the overlapping age categories, for example, study age range 15–45 would have been coded as ‘Adolescents’, ‘young adults’ and ‘adults’. In practice this means that a single record may appear in the map in multiple locations, making the evidence base appear to be larger than it is (see categorises below).

- Sectors
- Education
- Family and Community
- Health
- Retail
- Sport, culture, leisure
- Travel (including commuting)
- Work
- Other (please specify)
- None Specified
- Population/Recruitment Setting
- General public
- Hospital/Clinical Setting
- Schools
- University/colleges
- In isolation/quarantine
- Administrative data
- Mass gathering/event
- Other (please specify)
- Not specified
- Does the paper specifically focus on equity or differential impact of determinants on behaviour for different groups based on e.g. age, sex, SES etc?
- Yes
- No
- Study Design
  • Observational—Cross‐sectional
  • Observational—Longitudinal/cohort
  • Qualitative
  • Review
  • Intervention study
  • Other
- Age
- Children under 11
- Adolescents 12–18
- Young Adults 18–30
- Adults (30–70 or not specified)
- Adults over 70
- Not reported
- Behaviour categories
- Handwashing
- Masks/face covering
- Physical Distancing
- Social Distancing
- Isolation/quarantine
- Respiratory hygiene/etiquette
- Cleaning surfaces
- Avoiding t‐zone
- Composite Measures of relevant behaviours
- Determinants (broad categories and subcategories)
- Behaviour
- Past behaviour
- Intention
- Other protective behaviour
- Cognition
- behaviour
- disease
- Motivation
- Social
- Cognitive capacity
- Other
- Demographics
- Age
- Education
- Employment
- Ethnicity
- Geographic location
- Parental status
- Relationship status
- Religion
- SES
- Sex
- Multi‐generational households
- Caring responsibilities/carers
- Other
- Disease
- Status
- Proximity
- Other
- Emotions
- Disease
- Other
- Health status
- General
- At‐risk group
- Vaccine status
- Disability
- Other
- Information
- Info seeking/consuming
- Quality/source
- Messaging
- Knowledge
- Behaviour
- Disease
- Other
- Intervention
- Education
- Info campaign
- Other
- Other
- Beliefs
- Cultural
- Resources
- Social
- Time
- Other
- Did the study measure and report on the relationship between a malleable determinant and one or more behaviour of interest?
- Yes
- No
- Unclear
- Did the paper use a named theory of behaviour change?
- Yes ‐ extract name of theory/theories in info box
- No

#### 4.6.3 Quality appraisal

##### Systematic reviews

For the EGM, we appraised the methodological quality of systematic reviews with AMSTAR‐2 (Shea et al., 2017). One author completed the AMSTAR‐2 for each systematic review, and all were checked by a second author. Any discrepancies were discussed until a consensus was reached or, if necessary, referred to a third author to make a final decision.

Seven domains can critically affect the validity of a review and its conclusions (critical items 2, 4, 7, 9, 11, 13, and 15) (Shea et al., 2017). The study's overall quality rating is deemed ‘high’, if there is no more than one non‐critical weakness, ‘moderate’, if there is no critical weakness, but more than one non‐critical weakness, and ‘low’ if there is no more than one critical weakness, and with or without non‐critical weaknesses. Studies were deemed ‘critically low’, where there was more than one critical flaw.

The 16 items in AMSTAR2 cover:

1. PICOS in inclusion criteria,
2. Ex‐ante protocol,
3. Rationale for included study designs,
4. Comprehensive literature search,
5. Duplicate screening,
6. Duplicate data extraction,
7. List of excluded studies with justification,
8. Adequate description of included studies,
9. Adequate risk of bias assessment,
10. Report sources of funding,
11. Appropriate use of meta‐analysis,
12. Risk of bias assessment for meta‐analysis,
13. Allowance for risk of bias in discussing findings,
14. Discussion and analysis of heterogeneity,
15. Assessment of publication bias,
16. Report and potential source of conflicts of interest.

#### 4.6.4 Primary studies

We did not assess the quality of primary studies included in the map and is not a mandatory component in the development of EGMs. Risk of Bias of primary studies will be assessed formally as part of a series of subsequent systematic reviews that are being carried out as part of the CoHeRe project, looking at the associations between malleable determinants and specific protective behaviours. In addition, presenting quality assessments using different tools in the same EGM would have also be problematic for interpretation.

### 4.7 Analysis and presentation

The review team imported all relevant research to EPPI‐Reviewer software (Thomas, 2010) and all relevant data was extracted to generate a live, accessible and interactive map using EPPI‐mapper software and EPPI Visualiser tool.

### 4.8 Stakeholder engagement

The review questions were developed through consultation with the Behaviour Change Group formed in response to COVID‐19 by the Public Health Agency, Northern Ireland. The group consists of public health officials and academic experts.

In addition, we convened two advisory groups, the first consisting of international experts on evidence synthesis, behaviour change, public health and the second consisting of members of the public recruited through established fora for public involvement in science, this group was paid for their time in attending meetings and reading and offering feedback on documents.

The advisory groups were convened during the screening phase of the EGM process and before data‐extraction. The groups were provided with background information about the project and had an opportunity to ask questions of the research team. They were then asked to provide feedback on a draft of the map framework and the proforma for data extraction/coding of studies to be included in the EGM. As noted above as a result of their input we retained sector in the map framework and added equity, drawing on the PROGRESS‐PLUS guidance on equity in evidence synthesis. In addition, the advisory group also recommended that information was captured on multi‐generational households and cognitive ability, as a determinant of COVID‐19‐related behaviours.

## 5 RESULTS

### 5.1 Description of studies

#### 5.1.1 Results of the search

**Figure 1:**
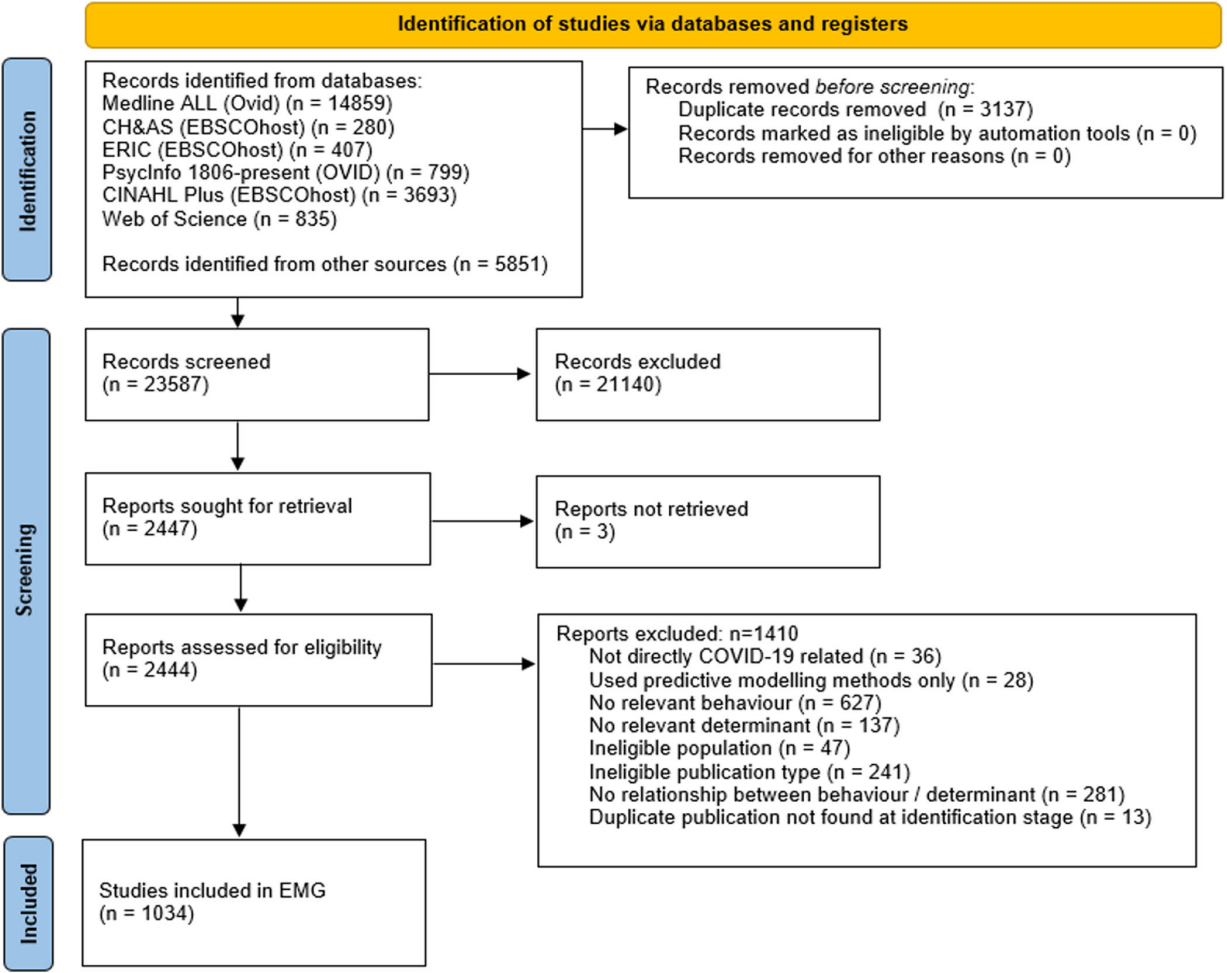
PRISMA flowchart for identification of studies included in the Evidence and Gap Map.

Our searches identified 26,724 records of which 3137 were duplicates. The 23587 unique records were screened by title and abstract, with 2444 screened at full‐text stage. We excluded 1410 studies at full‐text screening and during the mapping process stage. Studies were excluded if they were not directly COVID‐19 related; used predictive modelling methods only; did not measure any relevant behaviour or determinant; measured one or more relevant behaviour and a potential determinant but did not report any data on any relationship between a behaviour and determinant; included an ineligible population; or were an ineligible publication type (see Figure 1).

Following exclusions, 1034 studies were eligible for inclusion in the current version of the map. Of these, a large majority (860, 83%) were cross‐sectional studies. 68 longitudinal, 78 qualitative, 25 reviews, 62 intervention studies, and 39 other study designs (including mixed‐methods, and comparison studies).

#### 5.1.2 Excluded studies

A number of studies were excluded during the mapping stage (*n* = 106). Reasons for exclusion were primarily due to no reported data on the relationship between determinant and behaviour (*n* = 34), no behaviour measured (*n* = 32), ineligible publication type (*n* = 21), no determinant measured (*n* = 12), not directly COVID‐19 related (*n* = 5), ineligible population (*n* = 1), and used predictive modelling methods only (*n* = 1).

#### 5.1.3 Studies awaiting classification

From the 23,587 full‐text studies screened, 74 studies were coded as awaiting classification. These were largely trial records or studies that could not be located. These were identified as potentially eligible however the trial record did not provide sufficient data to determine eligibility.

### 5.2 Synthesis of included studies

Of the 1034 studies included in the current EGM the majority (n = 860: 83%) were cross‐sectional studies (Table 4). Of these, most were surveys conducted with the general population using retrospective self‐report measures of both behaviour and determinants.

**Table 4:** Included studies by study design.

Study design	Number of included studies
Cross sectional	860
Longitudinal/Cohort	68
Qualitative	78
Reviews	25
Intervention study	62
Other (e.g., mixed methods)	39

*Note*: See link to interactive map; https://eppi.ioe.ac.uk/eppi-vis/Review/Index.

#### 5.2.1 Where were studies conducted?

**Figure 2:**
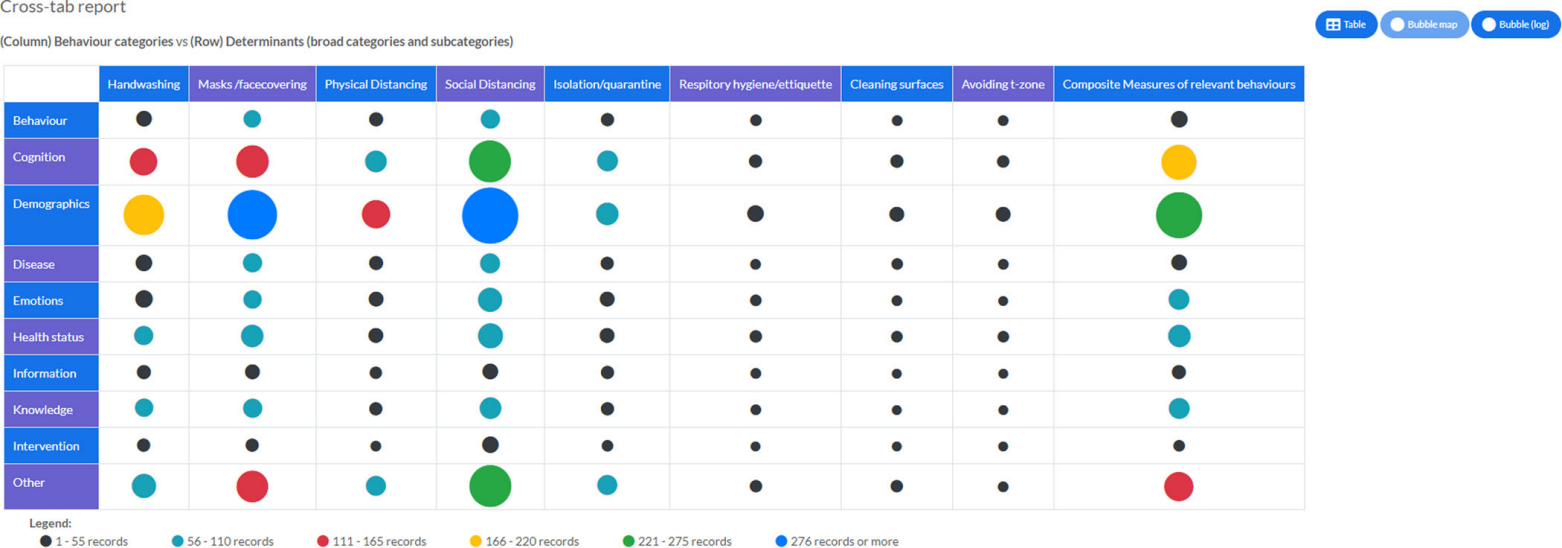
Numbers of studies included in the Evidence and Gap Map by behaviour and determinant categories.

**Figure 3:**
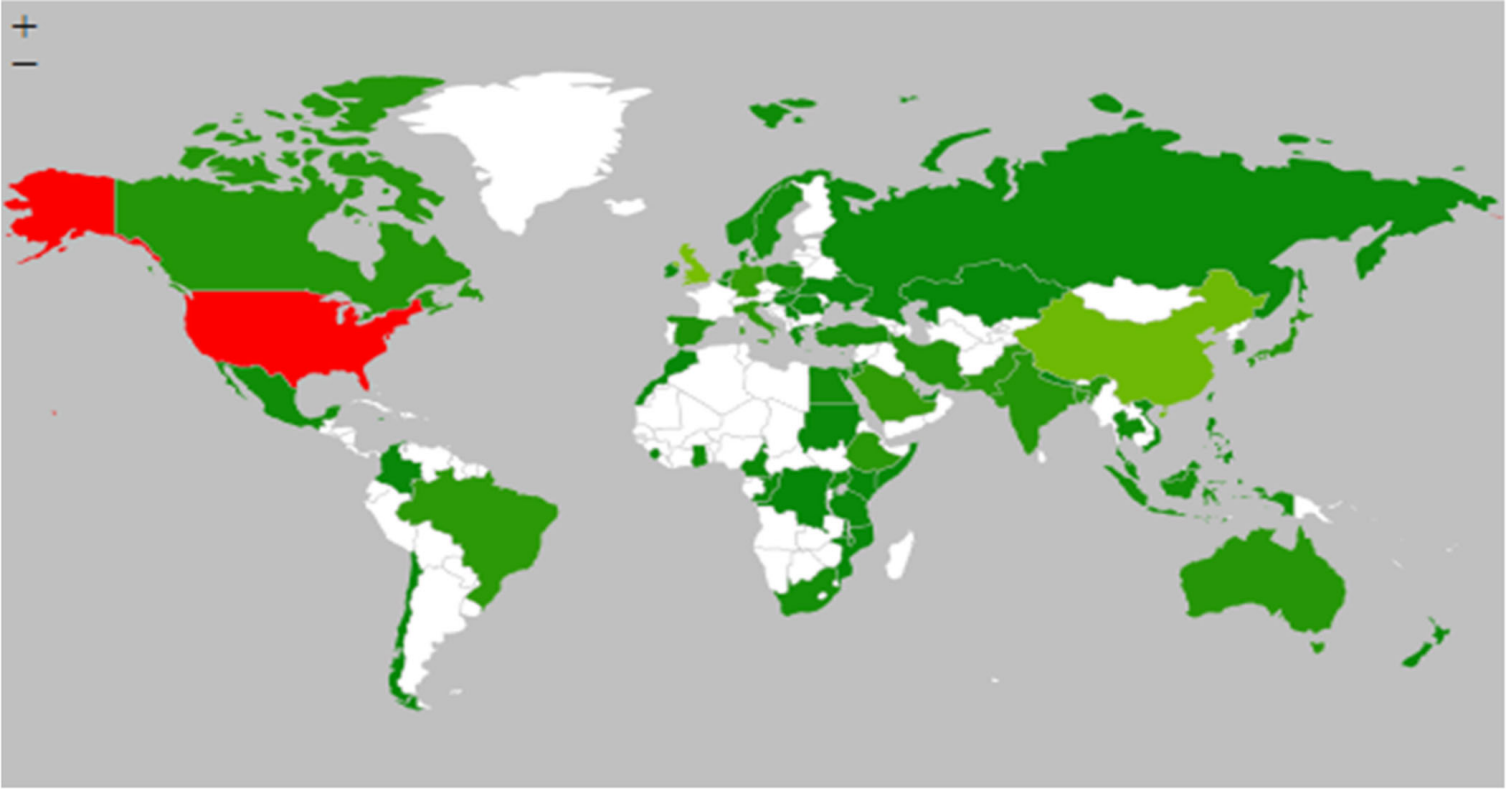
Geographical distribution of the studies included in the Evidence and Gap Map.

Figure 2 shows the number of studies included in this EGM by behaviour and determinant categories. The largest cluster of evidence came from North America (415 studies), followed by Asia (387 studies) and Europe (299 studies). Fewer studies came from Africa (118 studies), South America (45 studies), and Oceania (30 studies). Some studies (37 studies) examined behaviours and determinants across multiple countries (Figure 3).

#### 5.2.2 Study population

Most studies were carried out among the general public, with the second most common population being university students/faculty. Studies typically did not seek to assess behaviours in a particular sector or setting or did not specify, but of those that did education and health care settings were most common. Furthermore, studies typically included adults aged 18–70, with fewer studies examining adults over 70, or adolescents between 12 and 17. Children under 11 were the least studied age group.

#### 5.2.3 What behaviours were measured?

In relation to individual behaviours social distancing and masks or face‐coverings had the largest overall volume of studies, followed closely by handwashing. Overall, respiratory hygiene or etiquette, cleaning surfaces and avoiding T‐zone had the smallest volume of studies available. Of the 1034 studies in the EGM, 487 measured social distancing, 382 masks and face coverings, 308 handwashing, 177 physical distancing, 157 isolation/quarantine, 75 respiratory hygiene/etiquette, 59 cleaning surfaces, and 48 avoiding touching the T‐zone. Studies could be mapped under multiple behavioural categories (i.e., where one study measured multiple behaviours) so the counts given will add up to more than the total number of studies. There were 333 studies which used a composite measure of two or more behaviours of interest.

At the outset of this synthesis each recommended behaviours to mitigate human‐to‐human spread of COVID‐19 was defined, based on West et al. (2020) description (see Table 2). Throughout the mapping process it became evident that there was inconsistency in how the terms social distancing and physical distancing were used within the included studies. With the two terms often being used interchangeably. For example, some studies defined “maintaining the recommended distance from others when physically present” as social distancing, while others defined social distancing as a term which encompassed both maintaining the recommended distance and minimising social contact with those outside of your own household. To maintain consistency in the mapping process, the definitions presented in Table 1 were used to categorise behaviours in each study and mapped accordingly. Where a study used the term social distancing to mean both maintaining the recommended distance and minimising social contact, this was mapped as a composite measure.

#### 5.2.4 How were behaviours measured?

Composite measures varied in construction and composition. For example, some composite measures used single items for assessing adherence to two or more behaviours while others constructed multi‐item subscales for various behaviours and then combined subscales to provide an overall score for health protective behaviours in general. Thus, there was varied degrees of validity and reliability of composite measures used within these 333 studies. As with the composite measure, measures of individual behaviours also varied in their composition. For example, some studies opted to use a single item to measure a behaviour such as social distancing, whereas other studies employed several items to measure a single behaviour. We did not explicitly extract information on how behaviours were measured (e.g., retrospective self‐report, direct observation, diary methods, etc.), but our impression is that the majority of studies relied on retrospective self‐reports of behaviour and/or self‐reported intended behaviour. Very few measured behaviour through any form of direct observation.

#### 5.2.5 What determinants were measured?

Non‐malleable determinants were much more commonly measured than malleable determinants. This suggests that the evidence base overall is more likely to provide information on the targeting of public health messaging/interventions than to provide data that can inform the factors that could be influenced by such interventions.

The most commonly measured group of determinants was demographics (see Table 5). The largest volume of studies was observed between social distancing and demographics, specifically between social distancing and age and sex. This pattern was observed for all behaviours of interest, with demographics making up the largest volume of studies for each individual behaviour and composite measures. Fewer studies were observed for demographics such as parental status, religion, caring responsibilities, and multi‐generational households. An “other” category was used to capture information that did not fit within the predefined subcategories. The vast majority of studies mapped under the demographic, other subcategory were studies examining political affiliation and health‐related behaviour.

**Table 5:** Total number of studies by behaviour and determinant.

Behaviour (*n*)										
Determinant (*n*)	Total	Handwashing	Masks/Face covering	Physical distancing	Social distancing	Isolation/quarantine	Respiratory hygiene/etiquette	Cleaning surfaces	Avoiding t‐zone	Composite measure
Behaviour	150	44	60	32	70	26	14	8	12	49
Cognition	499	130	162	90	231	80	30	29	22	182
Demographics	731	217	281	132	330	93	53	43	40	259
Disease	157	52	69	34	78	29	13	14	9	47
Emotions	225	55	63	40	107	42	16	11	6	83
Health Status	239	72	96	42	109	43	22	17	15	91
Information	103	35	39	24	47	26	9	7	4	36
Intervention	98	30	31	13	49	15	7	7	6	15
Knowledge	192	61	67	30	85	27	9	6	4	81
Other	450	106	157	78	226	75	20	24	11	142

Following demographics, cognitive factors were the next most commonly measured determinants. Cognition regarding the behaviour and/or disease had the largest overall volume of studies, with perceived susceptibility and perceived risk being the most commonly measured determinant in these sub‐categories. Within the category of cognition, cognitive capacity had the fewest studies for each behaviour. Studies that measured cognitive capacity examined factors such as Intelligence Quotient (IQ), reading ability and health literacy.

Following demographics and cognition, the third‐largest volume of studies were categorised as“other” determinant category. This category included the subcategories of, beliefs, social, resources, other, time, and cultural. The subcategory of other (i.e., other, other) had the largest volume of studies mapped among this category. Studies mapped within this subcategory examined determinants such as; personality traits such as self‐efficacy, conscientiousness, emotional stability, agreeableness, narcissism, psychopathy, beliefs such as trust in government and conspiracy theories in relation to the COVID‐19 pandemic. Few studies were mapped in relation to cultural determinants such as collectivist or individualist cultures.

Relatively few studies measured past behaviour, behavioural intention, and other protective behaviours as a determinant of health‐related behaviours. In addition, few studies measured information seeking, information sources and messaging as a determinant. The fewest studies were observed for interventions as a determinant of health‐related behaviours. Within this category, we identified educational and information campaign interventions as well as ‘other’ intervention types including state‐wide mandates and policy. These studies were largely derived from the USA.

### 5.3 Risk of bias in included reviews

**Figure 4:**
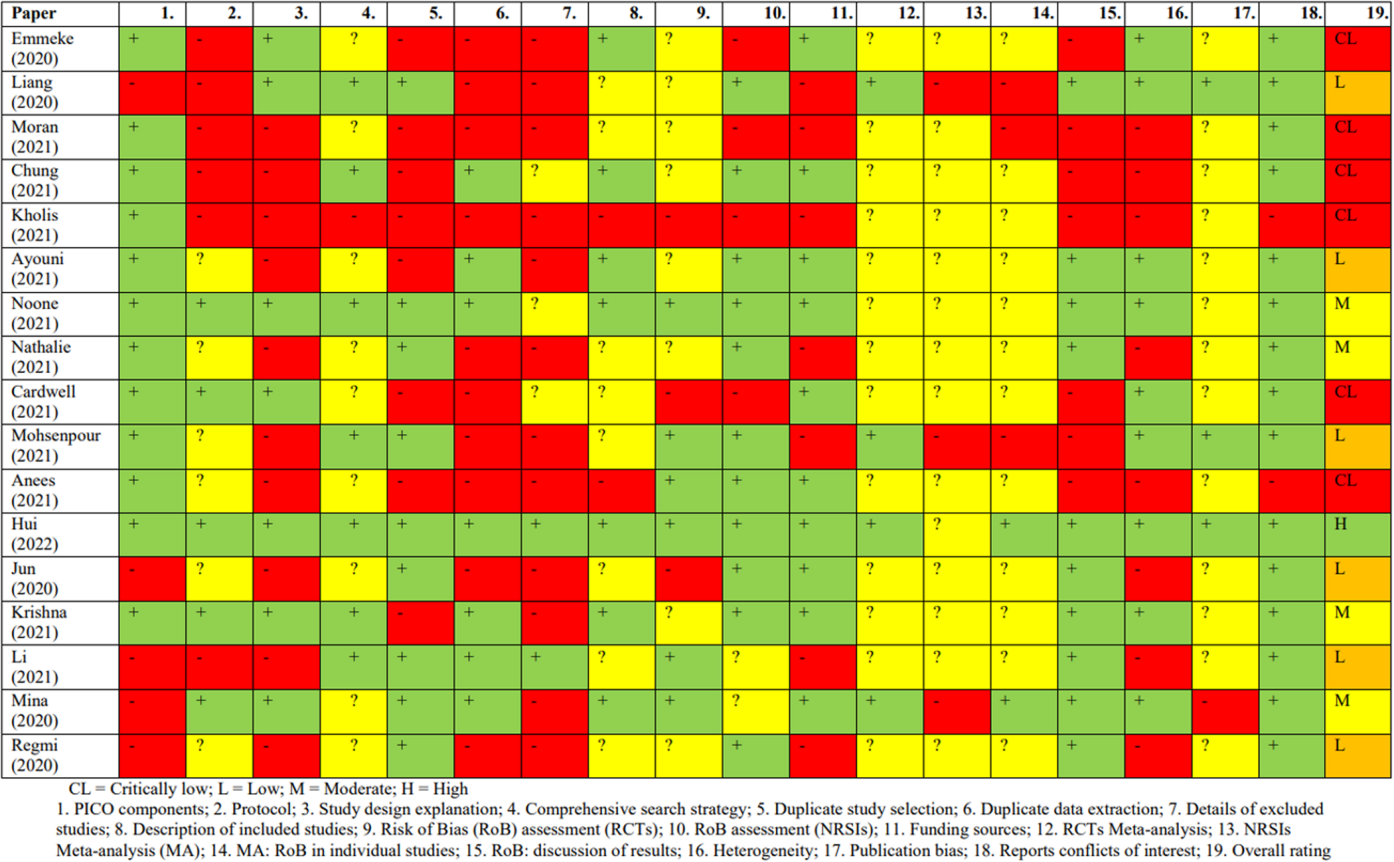
Risk of bias of included systematic reviews assessed using the AMSTAR‐2 (Shea et al., 2017).

**Figure 5:**
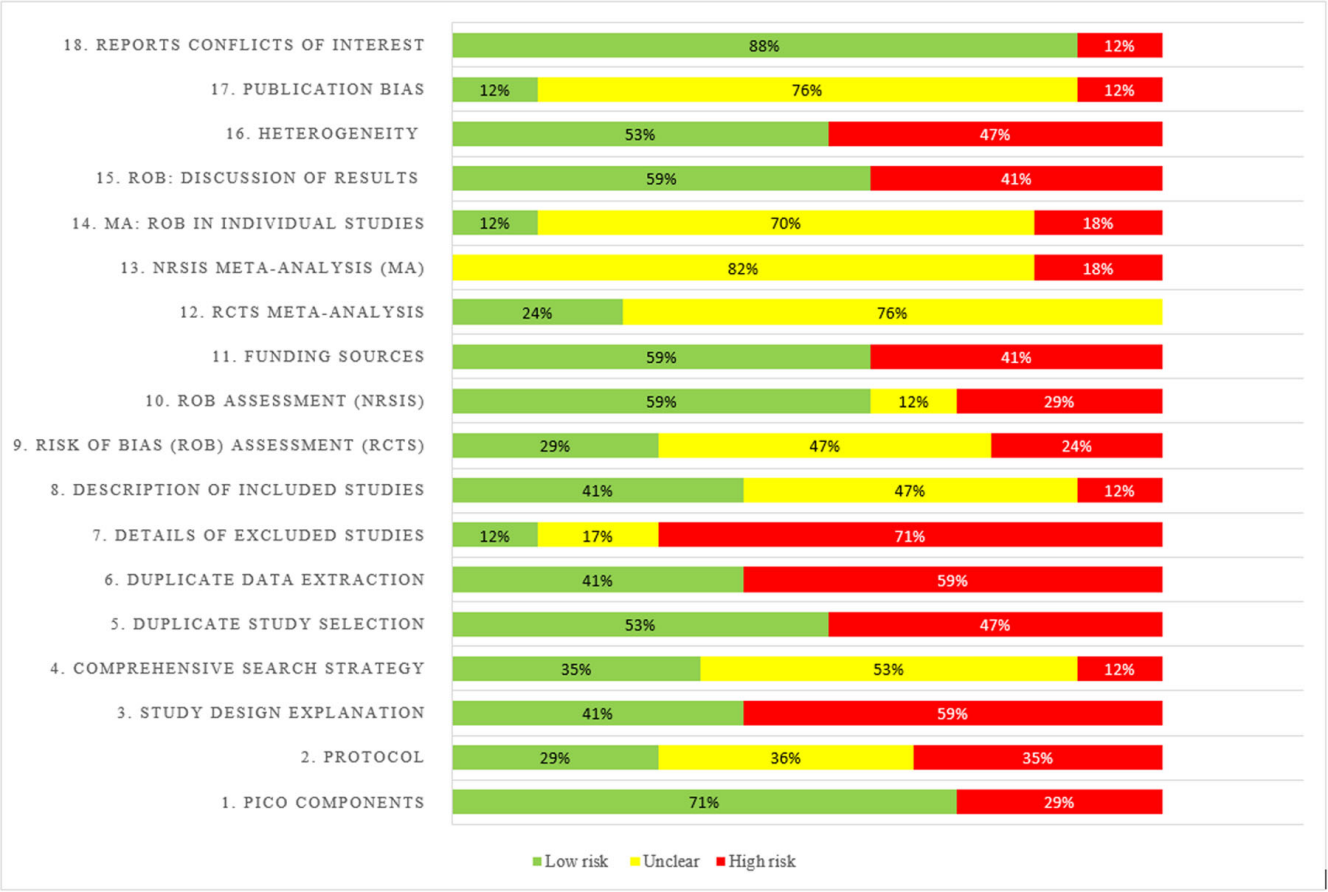
Summary table showing ratings for each item of the AMSTAR‐2 used to assess risk of bias in the included systematic reviews (Shea et al., 2017).

In total, 56 reviews were identified for possible inclusion. Of these, 39 were narrative reviews or were at protocol stage only. Only 16 of the remaining studies employed systematic search methods to identify included studies (including 14 systematic reviews and 3 scoping or rapid reviews). The quality of these studies were assessed using the AMSTAR−2 tool (Shea et al., 2017). One review was rated as high quality (Talic et al., 2021), three were moderate quality (Bakhit et al., 2021; Clavel et al., 2021; Noone et al., 2021), six were low quality (Ayouni et al., 2021; Jun et al., 2020; Li et al., 2021; Liang et al., 2020; Mohsenpour et al,. 2021; Regmi et al., 2021), and six were critically low (Anees et al., 2021; Cardwell et al., 2022; Chung et al., 2021; Emmeke et al., 2020; Kholis et al., 2021; Moran et al., 2021) (containing more than one ‘critical’ flaw) (Figures 4 and 5). Common flaws included lack of risk of bias assessment, failure to account for potential bias during interpretation of findings, unclear study inclusion and exclusion criteria, and failure to perform data extraction and screening procedures in duplicate or with adequate cross‐checking.

### 5.4 Additional dimensions

Additional filters were added to the EGM, such as age groups, study design, sector, population, and if the paper had a focus on equity.

Studies could also be filtered by whether the study was grounded in a behavioural theory. The majority of studies (*n* = 897) did not do so. Of those that did explicitly cite a named health behaviour/behaviour change theory the most commonly used theory was the Health Belief Model. Other theories included, the Theory of Planned Behaviour, Social Cognitive theory, Protection Motivation Theory, self‐determination theory, Social Norms Theory, and the theory of reasoned action.

## 6 DISCUSSION

### 6.1 Summary of main results

This report gives a detailed overview of an EGM, produced as part of the COHeRe project. The map summarises the body of evidence for studies examining psychological and psychosocial determinants of COVID‐19 health‐related behaviours. It provides a valuable resource for researchers, policy‐makers and the public to access the broad range of available evidence that has rapidly emerged since the beginning of the COVID‐19 pandemic. The map can be used to inform policy during the current pandemic and potential future outbreaks of respiratory infections with pandemic potential. It can also be used to help develop research questions in this important area of public health practice.

The current EGM version includes 1034 records, the vast majority of these relate to use of social distancing, masks or face coverings, and handwashing (between approximately 300‐500 records each). A substantial number of these (approximately 330 studies) assessed composite measures of behaviours. An important finding was that the largest cluster of studies examined non‐malleable determinants of protective behaviours. The vast majority of the determinants (examined in more than 700 studies) were demographic factors such as age and gender. Numerous studies also examined determinants relating to cognition, or people's thoughts, understanding or experiences in the context of COVID‐19 (approximately 500 studies). As with the behaviours, determinants categorised as ‘other’ were also common (approximately 450 studies). These included cultural factors, and issues around access to resources (such as availability of masks or face coverings). In terms of the design of the studies included in the EGM, most were online, cross‐sectional surveys (more than 850 of the 1034 studies). Another finding to note was that studies which examined the influence on behaviour of political affiliation, trust in government, and mandates, are largely derived from the United States. Only a small number of studies are focused on assessment of past behaviours, or behavioural intention, or on the role of other protective behaviours as determinants. The number of studies which are grounded in relevant behavioural theories is also limited.

### 6.2 Areas of major gaps in the evidence

Less evidence is available for some protective behaviours including physical distancing, and isolation or quarantine (between 150 and 180 records each). Even fewer studies (less than 75 records for each) assess respiratory hygiene/etiquette, surface cleaning or, avoiding touching the T‐zone. Relatively limited evidence was also found for some determinants, including those linked to interventional approaches, or the influence of individuals’ sources of information on COVID‐19. Views on, and the influence of maintaining other protective behaviours was also limited (between approximately 100–150 for each grouping).

Systematic reviews included in the EMG were assessed using the AMSTAR‐2 tool to provide an assessment of risk of bias in this evidence. The quality of these reviews was typically low. This was often due to failure to perform independent searches and data extraction procedures, as well as absence of clear explanations for study inclusion criteria, and lack of risk of bias assessment of the included evidence. Only five of 16 reviews were assessed as moderate or high quality. Future evidence synthesis studies should be improved by following relevant recommendations for the conduct of systematic reviews, particularly where evidence is required to be summarised rapidly in the context of emerging research.

### 6.3 Potential biases in the mapping process

To limit potential bias, a systematic approach, which included input from an information retrieval specialist, was used to plan and conduct the searches and the study identification process. Searches also included information sources such as trial registers and repositories, which were used to identify recent and rapidly emerging evidence. Other strengths include the extensive use of stakeholder involvement via advisory panel input, and through participation of the Cochrane Crowd, who contributed to the screening of a large number of potential records for inclusion.

### 6.4 Limitations of the EGM

Risk of Bias assessment is not a mandatory component in the development of EGMs, and given the large number of studies, this assessment was not carried for all studies. Risk of Bias of primary studies will be assessed formally as part of a series of subsequent systematic reviews that are being carried out as part of the CoHeRe project, looking at the associations between malleable determinants and specific protective behaviours. One important limitation is that the definitions used for some behaviours were inconsistent across much of the included evidence. The frequent use of composite measures may also limit the ability to examine the influence of determinants on specific behaviours.

### 6.5 Stakeholder engagement throughout the EGM process

There were no substantial changes with stakeholder engagement throughout the EGM process. All members of the advisory panel remained engaged with the EMG process throughout, and contributed to development of the mapping framework, including its content and visual presentation.

## 7 AUTHORS’ CONCLUSIONS

### 7.1 Implications for research, practice and/or policy

This report gives a detailed overview of an EGM, produced as part of the COHeRe project, that summarises evidence looking at determinants of COVID‐19 projective behaviours. The EMG is an accessible resource, which provides a summary of a large body of evidence, and which distinguishes malleable from non‐malleable factors. The map also identifies important gaps in the available evidence. This information can be used to inform prioritisation of research in this area.

The EGM included 1034 studies, of which 25 are reviews (including 17 systematic reviews) and 1009 are primary studies. Of the primary studies, 860 are cross‐sectional, 68 are longitudinal, and 78 are qualitative studies. The largest volume of evidence is observed for social distancing, face coverings, and handwashing. Comparatively, little evidence is available for other behaviours, including respiratory hygiene, cleaning surfaces and avoiding touching the T‐zone. In addition, the majority of studies examine eable determinants only, with demographic factors such as age making up the largest volume of evidence. This EGM categorised SES and employment status under the non‐malleable category of demographics. It is important to note that during the COVID‐19 pandemic these determinants were modified to some degree (i.e. furlough schemes). However, these were short‐term measures, and agruably non‐malleable through public health interventions.

There are a number of factors that may account for the patterns observed in the EMG. For example, the limited number of studies assessing some behaviours may reflect that these become less prominent over time, or following the early phases of the pandemic as new information about the virus and its transmission routes emerged. The finding that most studies used cross‐sectional methods might reflect that such study designs are an appropriate, but also more practical methodology for assessing individuals responses and perspectives on COVID‐19 mitigating behaviours, particularly given limitations on conducting face‐to‐face research during the initial stages of the pandemic.

Differences in adherence to protective behaviours may be due to static or relatively stable factors, such as demographic factors or personality traits. For example, increasing age, and conscientiousness might be associated with greater acceptance of COVID‐19 protective measures. While non‐malleable determinants could be used to target interventions at specific groups; malleable determinants, such as COVID‐19‐related concerns or fears, could potentially be directly influenced by interventions, including health messaging. It is still important to recognise that the more malleable determinants that have been identified in this map may also be relatively stable characteristics. These factors could also require some time to be influenced by interventional approaches designed to improve uptake of protective behaviours. Behaviours may also be influenced by the stage of the pandemic at which studies were conducted, and the mitigating factors that were in place at the time in that location. A recent series of cross‐sectional surveys, suggested that while motivation to engage with COVID‐19 protective behaviours may decrease over time, capability (including knowledge about transmission routes) and opportunity (such as public spaces being altered to support distancing behaviours) may increase (Smith et al., 2022).

There is a clear need for further research in this area. This should include in‐depth qualitative work, focusing on peoples’ views on the acceptability of interventions. This work should assess the extent to which recommended behaviours and interventions are considered to be appropriate and feasible; based on individuals’ anticipated or experienced cognitive and emotional responses. Further prospective longitudinal studies will also be required to evaluate changes over time related to important determinants of behaviours in response to COVID‐19, and to interventions aimed at influencing behaviours. Additional gap maps could also be produced in the future, focusing specifically on studies examining the impact and effectiveness of these interventions.

Evidence included in this EGM will be explored further through a series of systematic reviews examining the strength of the association between malleable determinants and the recommended behaviours. Findings from these reviews will be used to inform the development of public health messaging and other interventions aimed at improving uptake and maintenance of important protective behaviours.

## CONTRIBUTIONS OF AUTHORS

This EGM was undertaken by a team with substantial expertise in systematic reviews, health behaviour and infectious diseases. Professor Martin Dempster, Principal Investigator (PI) of the project had overall responsibility for its conduct and delivery. Dr Jennifer Hanratty was responsible for the day‐to‐day operation of the review, led screening, data extraction, quality assessment and reporting. Dr Ciara Keenan acted as an information retrieval specialist, designed and conducted the searches, and contributed to screening and data extraction. Dr Rachel Leonard contributed to screening, data extraction and drafted this report. Dr Sean O'Connor contributed to screening, data extraction and drafting this report. Ariana Axiaq, Yuan Chi and Janet Ferguson contributed to screening and data‐extraction. Professor Miller acted as advisor on evidence synthesis methodology. Dr Bradley was the content expert on communicable diseases and reviewed and commented on drafts.

Dr Jennifer Hanratty is a psychologist and expert in evidence synthesis. She has worked in evidence synthesis since 2012 and published reviews with Campbell, Cochrane and NIHR Health Technology Assessment among others, was editor with Campbell Education Co‐ordinating group, Fellow with Campbell UK & Ireland and an invited member of the international advisory board for Evidence Synthesis Ireland.

Dr Ciara Keenan is a methods editor and information retrieval specialist for the Campbell collaboration. She has considerable experience conducting and leading the creation of EGMs and systematic reviews.

Dr Sean O'Connor is a Physiotherapist and an experienced health care researcher. He has undertaken a number of systematic reviews and studies related to behavioural interventions, including in the context of COVID‐19. He has an extensive knowledge of theory‐based implementation models for maximising integration of evidence into practice, systematic review methods including methodological quality/risk of bias assessment and the examination of stakeholder perspectives in healthcare delivery.

Dr Rachel Leonard is a Social Worker and an experienced health care researcher. She has experience of conducting and leading on a number of systematic reviews, meta‐analyses, and studies related to health interventions.

Ariana Axiaq is a third year medical student with an interest in health promotion and extensive research experience in thematic analyses and systematic reviews.

Yuan Chi is Cochrane Information Specialist, Founder CEO of Yealth Technology, core team member with Cochrane COVID‐19 Study Register (https://covid-19.cochrane.org/), and the only Chinese executive team member for COVID19 Recommendation Map (https://covid19.recmap.org/). She has 8 years experience on evidence synthesis, assisted 50+ international projects, resulting in 11 authorships and 28 recommendations from Cochrane editors, information specialists and authors.

Dr Janet Ferguson is a researcher with a background in the use of technology to provide training in social communication to parents of autistic children. She has experience in assisting and conducting Systematic Literature Reviews across a number of related topics.

Professor Sarah Miller is Director of Campbell UK & Ireland. She is co‐chair and co‐editor of the Campbell Education Coordinating Group.

Dr Bradley is a consultant in public health medicine and clinical lecturer in public health and was consultant in health protection (communicable disease control) before taking up his current post. His publishing record includes several systematic reviews and studies of healthcare‐related behaviour. He is a member of the Northern Ireland COVID‐19 Modelling and Behaviour Change Groups

Professor Dempster, is a registered Health Psychologist and Chartered Statistician, with over 20 years’ experience in conducting research on the determinants of behaviour change. He has published 14 reviews, including reviews of effectiveness and reviews of covariates. He is currently a member of the Northern Ireland Public Health Agency COVID‐19 Behaviour Change Group.

○ Content: Bradley, Dempster, Hanratty, Miller, Keenan, O'Connor, Leonard
○ Systematic review methods: Hanratty, Miller, Dempster, Keenan, O'Connor, Leonard
○ Statistical analysis: Dempster, Miller, Hanratty, Keenan, O'Connor, Leonard, Ferguson, Axiaq, Chi
○ Qualitative Evidence Synthesis: n/a
○ Information retrieval: Hanratty, Keenan, O'Connor

## DECLARATIONS OF INTEREST

None of the review team have any present or past affiliations or other involvement in any organisation or entity with an interest in the EGM's findings that might lead to a real or perceived conflict of interest.

## PLANS FOR UPDATING THE EGM

The EGM will be updated on a rolling basis as new studies are identified up to the end of our funding period on 18th October 2022. Teams interested in building on the EGM or contributing to updating beyond the project end date are encouraged to contact the corresponding author.

## PUBLISHED NOTES

**Characteristics of excluded studies**

**Table:** 

Abbasi‐Kangevari, 2021
**Reason for exclusion**
Abdelhafiz, 2020
**Reason for exclusion**
Agusi, 2020
**Reason for exclusion**
Ainslie, 2020
**Reason for exclusion**
Al‐Shattarat, 2021
**Reason for exclusion**
Alexander, 2020
**Reason for exclusion**
Alhazmi, 2020
**Reason for exclusion**
Alhusseini, 2021
**Reason for exclusion**
Amaechi, 2020
**Reason for exclusion**
Aradhana, 2020
**Reason for exclusion**
Aynalem, 2021
**Reason for exclusion**
Bacon, 2021
**Reason for exclusion**
Bakdash, 2021
**Reason for exclusion**
Barry, 2021
**Reason for exclusion**
Bartels, 2021
**Reason for exclusion**
Bavel 2020
**Reason for exclusion**
Betsch, 2020
**Reason for exclusion**
Bhutta, 2020
**Reason for exclusion**
Block, 2020
**Reason for exclusion**
Blumer, 2021
**Reason for exclusion**
Bonati, 2021
**Reason for exclusion**
Carfora, 2021
**Reason for exclusion**
Cloosterman, 2021
**Reason for exclusion**
Collignon, 2021
**Reason for exclusion**
Commodari, 2020
**Reason for exclusion**
Cowling, 2020
**Reason for exclusion**
Cronin, 2021
**Reason for exclusion**
Dave, 2020
**Reason for exclusion**
de Bruin 2020
**Reason for exclusion**
de Bruin, 2020a
**Reason for exclusion**
Dubey, 2020
**Reason for exclusion**
Finkelstein, 2011
**Reason for exclusion**
Fisher, 2020
**Reason for exclusion**
Flowers, 2016
**Reason for exclusion**
Flowers, 2016a
**Reason for exclusion**
Foad, 2021
**Reason for exclusion**
Foad, 2021a
**Reason for exclusion**
Fodjo, 2020
**Reason for exclusion**
Fung, 2007
**Reason for exclusion**
Fung, 2007a
**Reason for exclusion**
Geldsetzer, 2020
**Reason for exclusion**
Goodwin, 2010
**Reason for exclusion**
Gosadi, 2021
**Reason for exclusion**
Guo, 2021
**Reason for exclusion**
Gupta, 2020
**Reason for exclusion**
Hager, 2020
**Reason for exclusion**
Hanson, 2021
**Reason for exclusion**
Harling, 2021
**Reason for exclusion**
Helsingen, 2020
**Reason for exclusion**
Hossain, 2020
**Reason for exclusion**
Isrctn, 2020
**Reason for exclusion**
Lardone, 2020
**Reason for exclusion**
Lawal, 2022
**Reason for exclusion**
Lazard, 2020
**Reason for exclusion**
Li, 2021
**Reason for exclusion**
Liang, 2020
**Reason for exclusion**
Lima‐Costa, 2020
**Reason for exclusion**
Lima‐Costa, 2020a
**Reason for exclusion**
Lin, 2020
**Reason for exclusion**
Lin, 2021
**Reason for exclusion**
Liu, 2021
**Reason for exclusion**
Madhu, 2020
**Reason for exclusion**
Mahdi, 2020
**Reason for exclusion**
Mallinas, 2021
**Reason for exclusion**
Miaskowski, 2021
**Reason for exclusion**
Mohsenpour, 2021
**Reason for exclusion**
New Study 1
**Reason for exclusion**
New Study 2
**Reason for exclusion**
New Study 3
**Reason for exclusion**
Nguyen, 2020
**Reason for exclusion**
Banholzer, 2021
**Reason for exclusion**
Ongan, 2020
**Reason for exclusion**
Pan
**Reason for exclusion**
Pettus‐Davis, 2021
**Reason for exclusion**
Prasad, 2020
**Reason for exclusion**
Prata, 2021
**Reason for exclusion**
Regmi, 2020
**Reason for exclusion**
Regmi, 2021
**Reason for exclusion**
Rezende 2021
**Reason for exclusion**
Richardson, 2020
**Reason for exclusion**
Rosenfeld, 2022
**Reason for exclusion**
Ryan 2021
**Reason for exclusion**
Saleh 2021
**Reason for exclusion**
Sarah
**Reason for exclusion**
Sari, 2020
**Reason for exclusion**
Siu, 2010
**Reason for exclusion**
Speaker, 2020
**Reason for exclusion**
Storch, 2020
**Reason for exclusion**
Tan, 2020
**Reason for exclusion**
Tao, 2020
**Reason for exclusion**
Thomas, 2021
**Reason for exclusion**
Tian 2020
**Reason for exclusion**
Toh, 2021
**Reason for exclusion**
Toomey, 2020
**Reason for exclusion**
Tsai, 2021
**Reason for exclusion**
van den, 2021
**Reason for exclusion**
van den, 2021a
**Reason for exclusion**
Vigurs, 2021
**Reason for exclusion**
Ward, 2020
**Reason for exclusion**
Ward, 2020a
**Reason for exclusion**
Weaver, 2021
**Reason for exclusion**
Webster, 2020
**Reason for exclusion**
Werneck, 2021
**Reason for exclusion**
Widhani, 2020
**Reason for exclusion**
Wolf, 2020
**Reason for exclusion**
Zou, 2021
**Reason for exclusion**

## SOURCES OF SUPPORT

**Internal sources**

- New Source of support, Other

**External sources**

- UKRI/ESRC Covid‐19 Research Award, Other

This project is supported by UKRI/ESRC Covid‐19 Research Funding. Further details are available here; https://gtr.ukri.org/projects?ref=ES%2FW002507%2F1.

## Supporting information

Supporting information.

Supporting information.
