# Supplementary material for: Psychological and psychosocial determinants of COVID Health Related Behaviours (COHeRe): An evidence and gap map
Source: Campbell Syst Rev. 2023 Jun 22;19(3):e1336. doi: 10.1002/cl2.1336 (PMC10286725; doi:10.1002/cl2.1336)
Supplement: Supplementary file 1 — Supporting information. [file CL2-19-e1336-s002.docx]

# Appendices

## 1 Description of determinant categories and subcategories

| **Determinants** | | |
| --- | --- | --- |
| ***Category*** | ***subcategory*** |  |
| Behaviour | Past behaviour | *Past behaviour as a determinant of current or intended behaviour* |
|  | Intention | *Behavioural intentions* |
|  | Other protective behaviour | *Papers that make links between doing one protective behaviour and another* |
| Cognition | behaviour | *Thoughts, perceptions about the protective behaviours -e.g. perceived efficacy, attitudes towards behaviours, perceived ease of engaging* |
|  | disease | *Thoughts/perceptions about the disease; e.g. perceived risk, severity, threat* |
|  | Motivation | *Motivation - anything related to motivations* |
|  | Other | *Other thoughts, perceptions, beliefs.* |
|  | Social | *Cognitions related to social factors; e.g. social norms, perception of other people’s behaviour etc.* |
|  | Cognitive capacity | *Studies where a person’s ability to understand and retain information about the behaviour, why it's recommended and how to engage in it. This code was added specifically as a result of discussion with our citizen partners and the challenges of caring for adults with severe memory loss or children with limited ability to understand and retain information about the behaviours.* |
| Demographics | Age |  |
| *only where data is presented on a relationship between behaviours of interest (see list above) and demographic variable(s).* | Education |  |
|  | Employment | *Employment status, occupation (income goes in SES)* |
|  | Ethnicity |  |
|  | Geographic location | *Rural vs urban, country etc.* |
|  | Parental status | *Is a parent or not, number of children in household etc.* |
|  | Relationship status | *Marital status, co-habiting status etc.* |
|  | Religion |  |
|  | SES | *socio economic status, includes income, household income, deprivation measures etc.* |
|  | Sex | *sex or gender* |
|  | Other | *e.g. political affiliation,* |
|  | Multi-generational households | *People living in multigenerational households or not.* |
|  | Caring responsibilities/ carers | *People who are responsible for caring for others (usually family members) but not in a professional capacity eg adults caring for their elderly parents, parents caring for children with disabilities or health challenges over and above the normal caring role of a parent. This code was added as a direct result of citizen advisor discussion on the responsibility for those caring for more vulnerable family members to reduce their risk of contracting and/or transmitting COVID in order to protect the person they care for but don't necessarily live with* |
| Disease | Status | *have symptoms, had it, recovered, positive test etc.* |
| *determinants related to things about COVID* | Proximity | *How close the disease is... e.g. local case rates, living in an affected area etc.* |
|  | Other | *Any other variables related to the disease, e.g. Time in the outbreak etc.* |
| Emotions | Disease | *Fear, worry, anxiety, concern for self or others but must be about the disease* |
| *feelings about the disease or general emotional state.* | Other | *Other feelings/emotional states/mood, e.g. general anxiety not focus on COVID, depressed mood. (Depression or anxiety that are conceived as clinical disorders/mental health issues would go under health)* |
| Health status | General | *Perceived health status, chronic condition* |
|  | At risk group | *People at particular risk of COVID for health reasons - e.g. underlying respiratory or immune condition. Apply a broad definition to include 'at risk' in terms of national guidance and those who self-define as at risk, due to for example autoimmune conditions or mental health challenges.* |
|  | Vaccine status |  |
|  | Other |  |
|  | Disability | *physical or intellectual disability. This code as added as a result of discussions with our citizen partners on disability being a potential barrier to people’s ability to understand or engage in the behaviours.* |
| Information | Info seeking/consuming | *Consumption of information, seeking information* |
|  | quality/source | *Quality of info, or the source of info* |
|  | Messaging | *Anything about the way information is presented - framing (gain framing, risk framing etc.), who delivers the message, content of messaging etc.* |
| Intervention | Education |  |
| *Broad category for studies that assess the effect of an intervention on behaviour* | Info campaign |  |
|  | Other | *add a little info here please as we may further divide this category if there are a lot of 'other' intervention approaches* |
| Knowledge | Behaviour | *Knowledge of protective behaviours, if they are effective, why they might be effective.* |
| *Typically assessed by a test of factual knowledge* | Disease | *knowledge of the disease - symptoms, transmission routes, case rates etc.* |
|  | Other | *Knowledge of other things e.g. how vaccines work, knowledge of local regulations etc.* |
| Other | Beliefs |  |
| *Any determinant that doesn't fit any other category* | Social | *social norms, social capital, social networks, social cues etc. this is likely to capture a mix of different concepts that may be unrelated to each other.* |
|  | Resources | *Access to practical resources e.g. paid sick leave, access to masks, ability to work from home, access to soap/sanitizer* |
|  | Other | *Anything that doesn’t fit neatly with other determinants* |
|  | Time | *Studies that specifically measure changes in the same people's behaviour over time* |
|  | Cultural | *Studies that use culture as a determinant - e.g. collectivist vs individualist cultures.* |

[Enter text here]

## 2 Medline (Ovid) search strategy

| **Appendix 2: Medline (Ovid) search strategy**  Ovid MEDLINE(R) ALL <1946 to September 03, 2021> | | |
| --- | --- | --- |
| 1 | SARS-CoV-2/ or COVID-19/ | 103591 |
| 2 | (corona* adj1 (virus* or viral*)).ti,ab. | 2364 |
| 3 | (CoV not (Coefficien* or "co-efficien*" or covalent* or Covington* or covariant* or covarianc* or "cut-off value*" or "cutoff value*" or "cut-off volume*" or "cutoff volume*" or "combined optimi?ation value*" or "central vessel trunk*" or CoVR or CoVS)).ti,ab. | 51911 |
| 4 | (coronavirus* or 2019nCoV* or 19nCoV* or "2019 novel*" or Ncov* or "n-cov" or "SARS-CoV-2*" or "SARSCoV-2*" or SARSCoV2* or "SARS-CoV2*" or "severe acute respiratory syndrome*" or COVID*2).ti,ab. | 181470 |
| 5 | or/1-4 | 187096 |
| 6 | limit 5 to yr="2020-Current" | 173962 |
| 7 | (6 and english.lg.) not (letter or historical article or comment or editorial or news).pt. not (Animals/ not humans/) | 134173 |
| 8 | (Mask or masks or face?mask* or Face cover*).ti,ab. | 42975 |
| 9 | (face adj2 (shield or shields)).ti,ab. | 414 |
| 10 | (((Hand or hands) adj2 hygiene) or Handwash* or (Wash* adj2 hand*)).ti,ab. | 11132 |
| 11 | (hand adj1 clean*).ti,ab. | 256 |
| 12 | (hand adj2 saniti*).ti,ab. | 683 |
| 13 | (hand adj2 disinfect*).ti,ab. | 783 |
| 14 | Respiratory hygiene.ti,ab. | 79 |
| 15 | Respiratory etiquette.ti,ab. | 27 |
| 16 | ((cough* or sneeze*) and (sleeve or arm or elbow or tissue or etiquette)).ti,ab. | 2752 |
| 17 | (tissue and (dispose or disposal or bin or hygiene)).ti,ab. | 3414 |
| 18 | universal hygiene.ti,ab. | 10 |
| 19 | Social Isolation/ or Patient Isolation/ | 19284 |
| 20 | (self-isolate or self-isolation or self-isolating).ti,ab. | 724 |
| 21 | (mass adj2 (behav* or gather*)).ti,ab. | 1690 |
| 22 | (social distance or social distancing).ti,ab. | 6625 |
| 23 | stay at home.ti,ab. | 1465 |
| 24 | stay home.ti,ab. | 314 |
| 25 | ((work* adj2 home) or telecommute or telework* or (remote* adj2 work*)).ti,ab. | 5262 |
| 26 | (Physical adj2 distanc*).ti,ab. | 2595 |
| 27 | (touch* and (mouth or mouths or face or faces or nose or noses or t-zone)).ti,ab. | 1635 |
| 28 | disinfect*.ti,ab. | 31760 |
| 29 | lockdown.ti,ab. | 8167 |
| 30 | quarantine.ti,ab. | 7821 |
| 31 | (nonpharmaceutical or non-pharmaceutical).ti,ab. | 1831 |
| 32 | (school closure or close school* or school closing).ti,ab. | 389 |
| 33 | or/8-32 | 140404 |
| 34 | limit 33 to yr="2020-Current" | 34955 |
| 35 | (34 and english.lg.) not (letter or historical article or comment or editorial or news).pt. not (Animals/ not humans/) | 31455 |
| 36 | 7 and 35 | 20298 |
| 37 | exp Knowledge/ | 12323 |
| 38 | exp Health knowledge, Attitudes, Practice/ | 119567 |
| 39 | (Knowledg* or Personal* or Attitude* or Practice* or Habit* or belie* or Behav* or Need* or prevent* or Compliance or comply* or complied or Perception* or Protect* or Predict* or view* or barrier* or facilitator* or readiness or prepar* or ability* or insight or proficien* or procedur* or adher*).ti,ab. | 10617318 |
| 40 | or/37-39 | 10635825 |
| 41 | 7 and 35 and 40 | 14859 |
