# Supplementary material for: Psychological and psychosocial determinants of COVID Health Related Behaviours (COHeRe): An evidence and gap map
Source: Campbell Syst Rev. 2023 Jun 22;19(3):e1336. doi: 10.1002/cl2.1336 (PMC10286725; doi:10.1002/cl2.1336)
Supplement: Supplementary file 2 — Supporting information. [file CL2-19-e1336-s001.docx]

**Abbreviations and acronyms**

EGM: Evidence and Gap Map
